# Editorial: Rising stars in veterinary epidemiology and economics 2022: Porcine Reproductive and Respiratory Syndrome Virus: Epidemiology, immunology and virology

**DOI:** 10.3389/fvets.2023.1111668

**Published:** 2023-01-19

**Authors:** Igor A. D. Paploski, Andreia G. Arruda, Kimberly VanderWaal

**Affiliations:** ^1^Department of Veterinary Population Medicine, University of Minnesota Twin Cities, St. Paul, MN, United States; ^2^Department of Veterinary Preventive Medicine, The Ohio State University, Columbus, OH, United States

**Keywords:** Porcine Reproductive and Respiratory Syndrome Virus (PRRSV), epidemiology, Editorial, swine, review

This Research Topic gathers different contributions highlighting important aspects of Porcine Reproductive and Respiratory Syndrome Virus (PRRSV) research, including the detection of multiple viral lineages within farms, the use of processing fluids for PRRSV detection in breeding herds, the emergence of a new PRRSV strain in China and the genetic diversity of PRRSV in Hungary. The topics covered by these papers are of emerging importance in the field. To evaluate the potential contribution that this Research Topic brings to the PRRSV research field, we downloaded all entries and citation metrics from PubMed for the term “Porcine Reproductive and Respiratory Syndrome Virus.” A total of 4,066 papers were identified. For each year between 2002 and 2021, we selected the five papers that accrued most citations since their publication, reviewed their abstracts, and tabulated the topics those papers covered.

Out of the 100 papers reviewed, 42% presented findings on PRRSV immunity; 37% on PRRSV genetics; 23% were on infection dynamics/epidemiology; 20% discussed findings on specific genes or proteins encoded by the PRRSV genome; 15% dealt with virus characterization; 10% on PRRSV classification; 8% on clinical manifestation after PRRSV infection; 4% on PRRSV risk factors; 3% on economic impact of PRRSV; and 24% were reviews (please note that the total is more than 100% because one paper could be classified in multiple categories). A list of the reviewed papers assigned to the six most frequent categories can be found on Figure 1. It is important to highlight that the list of 100 reviewed papers above does not include all published papers in the period, only the top-five most cited papers in each of the last 20 years. Our analysis showed that a lot of attention has been given to understanding the mechanisms behind the immune response of PRRSV, and perhaps rightfully since the control of many diseases historically has been achieved through a better understanding of how hosts respond to infection and how that can be leveraged to achieve protection, for example, *via* vaccination. Understanding the genetic differences between viruses has also been a heavily cited field of the reviewed papers. Interestingly, not a large fraction of the top-cited papers were related to epidemiology.

**Figure 1:**
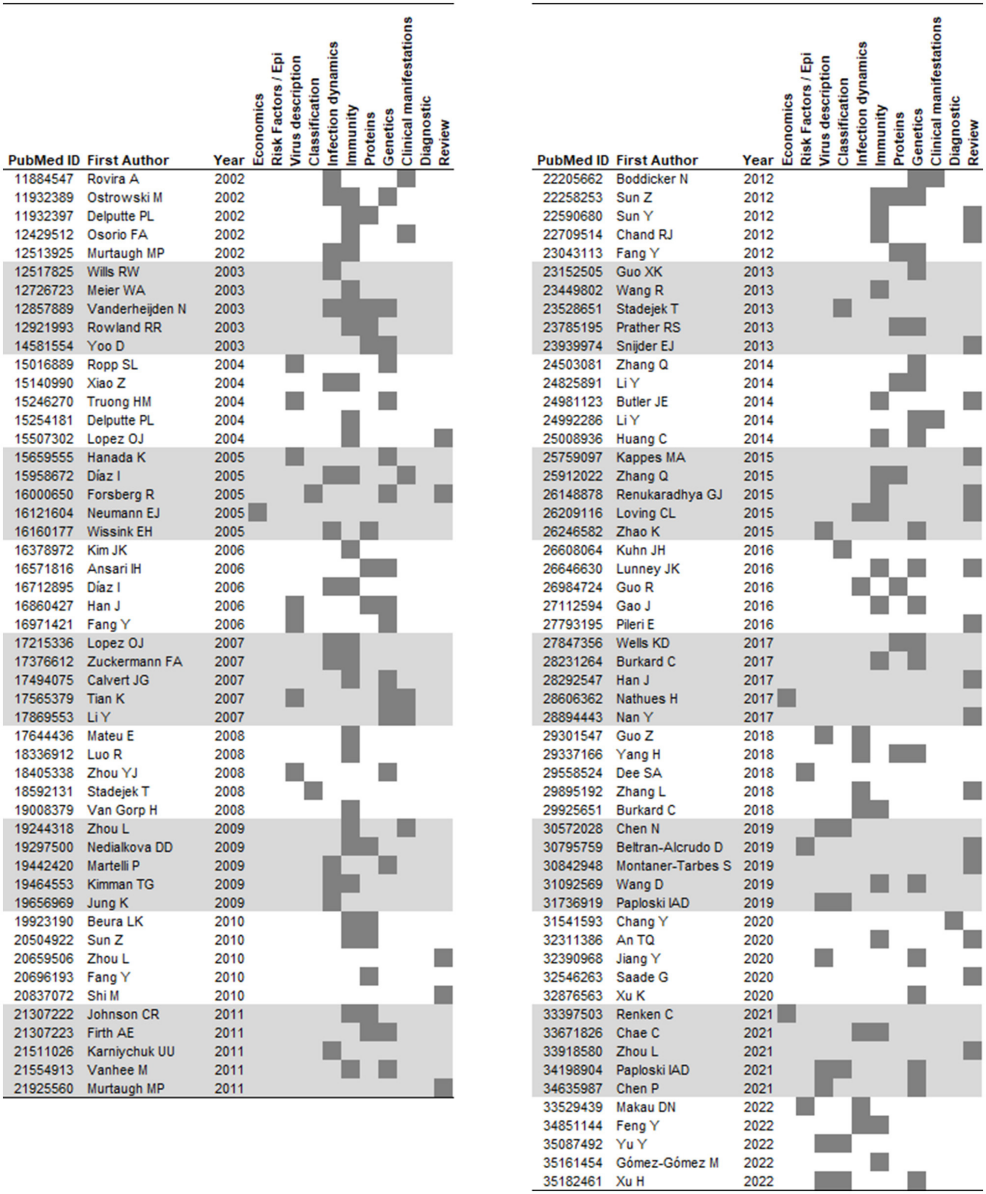
List of top-five most cited papers per year (2002–2022) according to the topic covered by the paper (gray boxes).

In this Research Topic, we present four papers that explore topics that have been under-represented in PRRSV literature. The paper by Cheng et al. reports the detection of multiple variants of PRRSV in breeding and growing swine herds. This is an overlooked issue, as usually when PRRSV sequencing is performed, a single (consensus) sequence is generated, and it is assumed that a single PRRSV variant is causing the outbreak on the farm. Cheng et al. elegantly demonstrated that this may not always be true and the consequences are important: conclusions of potential routes of introduction of PRRSV in a farm may be misleading if complete knowledge about PRRSV variants circulating in contacts of a farm is absent. Additionally, planned intentional exposure efforts (such as live virus inoculation or vaccination) that are informed based on which viruses circulate on a farm may be driven based on incomplete and potentially inadequate data.

The paper presented by Kikuti et al. presents the potential impact that evaluating PRRSV presence on a farm by using processing fluids may have on the trends of PRRSV occurrence over time. Adoption of processing fluid testing increased mostly after 2017, and its ability to test a larger number of animals with an assumed adequate sensitivity means that the industry can more accurately evaluate if and for how long PRRSV is still circulating on a sow farm after an outbreak. This means that farms now may take longer, on average, to reach a negative status after an outbreak. This is not necessarily because of the virus' characteristics, but because our ability to detect if the virus continues to circulate on a farm has changed. This changes how information on PRRSV circulation should be interpreted, particularly in involving periods before and after the wide adoption of this sampling strategy.

Sun et al. report on a recombinant PRRS virus found in China. The recombinant parents of the virus found in China are two vaccine viruses. Recombination, the mixing of two PRRSV genomes into a novel PRRSV, may potentially occur when two viruses infect the same cell. The virus resulting from recombination can be antigenically unique or have a different virulence, both of which could lead to a change in the clinical disease, but the exact consequences of a recombination event are hard to anticipate. In this paper, one of these vaccines deemed to be the recombinant parent of the virus reported is not authorized for use in China. This raises concerns about how porous borders may be, which in itself presents a large challenge, as the recent African Swine Fever massive outbreak in China demonstrates.

The last paper of the Research Topic, by Jakab et al. reports on the genetic diversity of PRRSV-2 sequences found in Hungary, highlighting the similarity between Hungarian viruses and those circulating in neighboring countries in the European Union. Even though the phylogenetic history of the strains found in Hungary was not assessed in the analysis, results suggest the possibility that PRRSV-2 vaccine-derived viruses, which use is authorized only in neighboring countries, may have entered Hungary. This represents a challenge, particularly given the goal that Hungary has of eliminating PRRSV from their swine herd.

Taken together, these papers contribute to sub-fields of the PRRSV literature that are relevant but that perhaps have not gained enough attention in the past 20 years. We hope that the reader will find this Research Topic a useful reference for PRRSV epidemiology.

## Author contributions

IP: conceptualization, analysis, writing—original draft preparation, and writing—review and editing. AA: writing—review and editing. KV: conceptualization and writing—review and editing. All authors contributed to the article and approved the submitted version.

